# Multispectral Image Compression Based on DSC Combined with CCSDS-IDC

**DOI:** 10.1155/2014/738735

**Published:** 2014-07-07

**Authors:** Jin Li, Fei Xing, Ting Sun, Zheng You

**Affiliations:** ^1^Department of Precision Instrument, The State Key Laboratory of Precision Measurement Technology and Instruments, Tsinghua University, Beijing 100084, China; ^2^Collaborative Innovation Center for Micro/Nano Fabrication, Device and System, China

## Abstract

Remote sensing multispectral image compression encoder requires low complexity, high robust, and high performance because it usually works on the satellite where the resources, such as power, memory, and processing capacity, are limited. For multispectral images, the compression algorithms based on 3D transform (like 3D DWT, 3D DCT) are too complex to be implemented in space mission. In this paper, we proposed a compression algorithm based on distributed source coding (DSC) combined with image data compression (IDC) approach recommended by CCSDS for multispectral images, which has low complexity, high robust, and high performance. First, each band is sparsely represented by DWT to obtain wavelet coefficients. Then, the wavelet coefficients are encoded by bit plane encoder (BPE). Finally, the BPE is merged to the DSC strategy of Slepian-Wolf (SW) based on QC-LDPC by deep coupling way to remove the residual redundancy between the adjacent bands. A series of multispectral images is used to test our algorithm. Experimental results show that the proposed DSC combined with the CCSDS-IDC (DSC-CCSDS)-based algorithm has better compression performance than the traditional compression approaches.

## 1. Introduction

Remote sensing multispectral images are obtained by optical multispectral camera carried on the satellite imaging multiple contiguous narrow bands of the same objects [1, 2].

In general, there are dozens of bands to a few ones in the wavelength range from visible to near infrared spectrum, and the spectral resolution of multispectral is 0.1*λ*, such as multispectral images produced PLEIADES satellite, IKONOS satellite, and QuickBird satellite. They can obtain abundant spatial and spectral information of measured objects simultaneously. Multispectral imaging technique has been widely applied in many fields, like science research, airborne and airspace remote sensing, medical devices, environment monitoring, geological survey, agricultural monitoring, military applications, and so on [3, 4]. Another efficient method for collecting images of an object in a series of spectral windows is hyperspectral imaging. Hyperspectral images have narrower but more number of bands. Typical applications of hyperspectral imaging approach also appeared on science research, airborne and airspace remote sensing, and military reconnaissance [5, 6]. In general, hyperspectral images consist of more finely divided spectral channels than multispectral images. However, hyperspectral images have lower spatial resolution than multispectral images. Multispectral images can sometimes refer to a set of images taken at vastly different parts of the electromagnetic spectrum. In this paper, we mainly research how to compress multispectral images using an algorithm having low complexity, high robust, and high performance according to characteristics of multispectral images.

With the development of remote sensing multispectral camera, its performance requirements, such as the field of view, resolution, and wide swath, are continuously improved. At the same time, photodetectors read rate and adopted AD quantization bits are also growing and improving. Finally, the captured digital image data are increased rapidly. However, because of the limited solid-state storage capacity on board and restricted satellite downlink channel bandwidth, it is difficult to adapt to the huge amounts of data of multispectral image. So, multispectral image data must be compressed.

Most multispectral image compression algorithms are based on a 3D transform [7] approach. The transform-based approach usually has an image transform stage. The transform approaches have block-based DCT [6], multiresolution scheme DWT [8], postwavelet transform, KLT or PCA [9], and so on. Then, the transform coefficients are coded by compression algorithms, such as EZW [10], SPIHT, SPECK, EBCOT [8], bit plane encoder (BPE) recommended by CCSDS-IDC, and so on. Recently, the typical transform-based algorithms are JPEG2000 [11] and remote sensing panchromatic images compression algorithm CCSDS-IDC [12]. In JPEG2000 algorithm, EBCOT is very efficient to remove the redundancy between wavelet transforms coefficients, which makes JPEG2000 become the best-performing compression encoder in the existing image compression algorithms. However, it is too complex to be implemented in space mission. The CCSDS-IDC algorithm is composed of DWT and BPE. The BPE, a zero tree encoder, makes most of the structures of spatiotemporal orientation trees in bit plane. That is, grandchildren coefficients also become not important when children coefficients are important. This zero tree characteristic makes the bit plane exit in a large amount of zero area. The coding efficiency can be improved by taking full advantage of this zero area can improve coding efficiency. CCDS-IDC has progressive coding and fault-tolerant capability characteristic. But also, BPE is of low complexity and occupies less storage capacity, which is very suitable for the application of on-board camera. However, it decreases the average PSNR by 2 dB compared with JPEG2000. In addition, CCDS-IDC is only suitable for the 2D image, which cannot exploit the spectral redundancy for 3D image.

In this paper, we propose an effective multispectral image compression method based on DSC combined with CCSDS-IDC by deep coupling way. The proposed algorithm has low complexity, high robust, and good performance, which is well suitable to application requirements of the new generation of high-resolution multispectral camera with wide field.

The remainder of this paper is organized as follows. In Section 2, we present the idea of the proposed CCSDS-DSC compression algorithm for multispectral images. In Section 3, we perform the verification experiments on multigroup multispectral images and analyze the experimental results.  Section 4 concludes the proposed algorithm and presents further scope for modifications that can be incorporated.

## 2. Proposed Scheme

To weigh the computational complexity and compression performance, in this paper, we proposed a multispectral images compression scheme, which reduces the computation complexity and is easy to hardware-implement without sacrificing spatial and spectral information. We integrate improved CCSDS-IDC and distributed source coding by deep coupling way to remove the spatial redundancy, spectral redundancy, and bit information redundancy. The proposed compression encoders are less extensive than traditional ones, which is suitable for the on-board camera application.

### 2.1. Improved CCSD-IDC Algorithm

**Figure 1:**
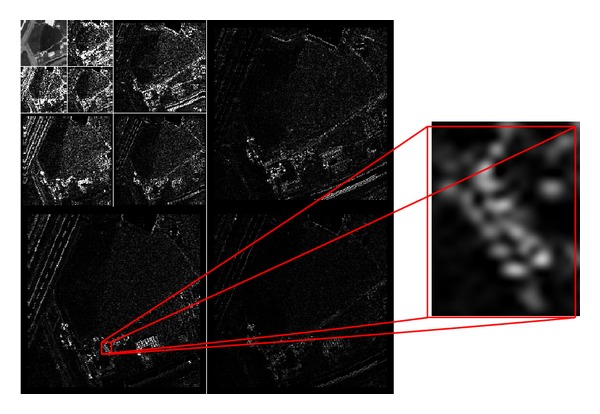
The wavelet transform of high resolution remote sensing image with a zoom on some coefficients.

In CCSDS-IDC algorithm, a 3-level 9/7 lifting 2D DWT is performed to remove the spatial redundancy. The 2D DWT can decompose the image into lower resolution and detail subband. The decomposing process is also viewed as multilevel low-pass filtering and high-pass filtering. An each level, the low resolution subband produced by the previous level decomposition is processed by the high-pass filter to produce wavelet coefficients and by the low-pass filter to produce the approximate information called scaling coefficients. However, after each band of remote sensing, multispectral images are transformed by the 2D DWT; the residual directional spatial correlation between wavelet coefficients in a small adjacent area still existed (see Figure 1). These residual directional correlations produce a large number of large amplitude high-frequency coefficients, which are disadvantage for the later coding.

**Figure 2:**
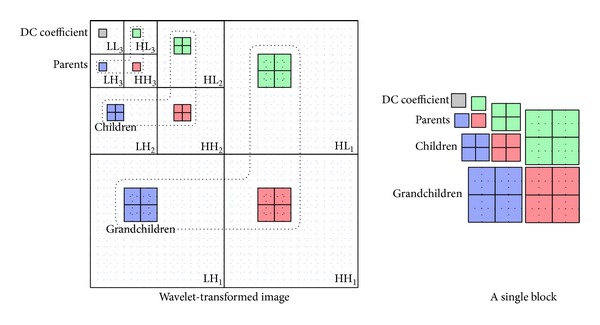
A coefficients block having 64 coefficients from different levels, and the 64 coefficients are all decomposed coefficients of a small adjacent area in original band of multispectral images.

In this paper, we apply a Hadamard transform (HT) to wavelet coefficients to remove residual redundancy between adjacent wavelet coefficients. According to CCSDS-IDC algorithm, three levels of two-dimensional wavelet decomposition are performed to 10 subbands, which are denoted as LL, *LH*⁡_*i*_, HL_*i*_, HH_*i*_, and *i* = 1, 2, 3. The BPE encodes wavelet-transformed coefficients by using blocks as basic processing units. Each block is composed of 64 coefficients having one DC coefficient and 63 AC coefficients, which is shown as in Figure 2. The DC coefficient is one of the scaling coefficients decomposed in the maximum level, and 63 AC coefficients are wavelet coefficients obtained in the level. It is noted that each block corresponds exactly to a small adjacent area in the original image.

In this paper, we apply HT to each subband of each single block. In grandchildren block, 4 × 4 HT is performed. In children block, 2 × 2 HT is performed. Each wavelet coefficients block is denoted by *F* that contains 4 or 16 coefficients which are denoted by *M* (*M* = 4 or 16) vector elements. *F* can be regarded as a vector which is composed of *M* elements in *R* ^*M*^. A HT orthonormal base is denoted by *B*_*b*_, which is composed of *M* base vectors, where *b* is a sequence number of subbands in single wavelet coefficients block, *b* ∈ [1,6]. Each base vector is denoted by *ϕ*_*m*_^*b*^, *m* ∈ [1, *M*]. So, the orthonormal base can be expressed as

(1) Bb={ϕmb}m=1M.

The HT-transformed coefficients block is denoted by *f* ^*b*^. The HT-transform of one 4 × 4 or 2 × 2 wavelet coefficients block can be expressed as follows:

(2) $$\begin{array}{c} f^{b}=\overset{M}{\underset{m=1}{\sum }}\langle f,\phi_{m}^{b}\rangle \cdot \phi_{m}^{b}. \end{array}$$

After each block projected on HT bases, one HT-transformed block and one original coefficients block can be obtained. The best-transformed coefficients block needs to be determined by an evaluation function based on the minimum rate-distortion (*R*-*D*) Lagrangian cost, which can be expressed as follows:

(3) $$\begin{array}{c} L\left(f_{q}^{b}\right)=D\left(f_{q}^{b}\right)+\lambda \cdot R\left(f_{q}^{b}\right), \end{array}$$

where *f*_*q*_^*b*^ denotes the quantized HT-transformed coefficients when quantization step is *q*, *λ* is a Lagrangian multiplier, which can be determined as

(4) $$\begin{array}{c} \lambda =\frac{3q^{2}}{4\gamma_{0}},\;\text{with}\;\gamma_{0}=7, \end{array}$$

and *D*(*f*_*q*_^*b*^) is the square error (SE) between *f* ^*b*^, which is the coefficients obtained by HT, and *f*_*q*_^*b*^,which is *f* ^*b*^ quantized at a quantization step *q*; that is,

(5) $$\begin{array}{c} D\left(f_{q}^{b}\right)={||f^{b}-f_{q}^{b}||}^{2}=\overset{M}{\underset{m=1}{\sum }}{\left|a_{b}\left[m\right]-Q_{q}\left(a_{b}\left[m\right]\right)\right|}^{2}. \end{array}$$

*R*(*f*_*q*_^*b*^) is the need bit rate to encode *f*_*q*_^*b*^, which can be expressed as

(6) $$\begin{array}{c} R\left(f_{q}^{b}\right)=R_{C}\left(f_{q}^{b}\right)+R_{b}, \end{array}$$

where *R*_*C*_(*f*_*q*_^*b*^) is the need bit rate to encode *f*_*q*_^*b*^, which can be estimated by

(7) $$\begin{array}{c} R_{C}\left(f_{q}^{b}\right)=\overset{M}{\underset{i=1}{\sum }}\log_{2}\frac{1}{p\left(f_{q}^{b}\right)}. \end{array}$$

*R*_*b*_ is the overhead of bit rate in order to encode the identification best base index b*, which can be expressed as

(8) $$\begin{array}{c} R_{b}=-\text{lo}{\text{g}}_{2}\text{Pr}\left(b\right),\;\;\text{Pr}\left(b\right)=\{\begin{array}{cc} 0, & b=0,\\ \frac{0.5}{N_{b}}, & b\in \left[1,N_{b}\right]. \end{array} \end{array}$$

One wavelet coefficients block carries out the HT-transform algorithm, which is shown as in Algorithm 1.

### 2.2. CCSDS-DSC Strategy

**Figure 3:**
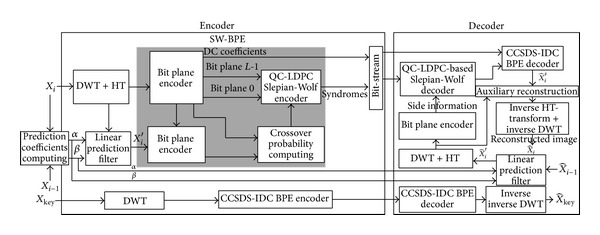
Proposed compression algorithm based on CCSDS-IDC merged to DSC by deep coupling way for multispectral images.

Figure 3 shows the proposed CCSDS-DSC entropy encoding algorithm. The key spectral band *X*_key_ is sparsely represented by wavelet transform and HT-transform. The transformed coefficients are encoded and decoded independently by CCSDS-BPE. The first spectral band is considered as the key band, which is used to predict for encoding the second spectral band and to reconstruct the second spectral band. The other spectral band *X*_*i*_ is predicted from the previous band *X*_*i*−1_. *X*_*i*_ is firstly HT-transform in wavelet domain, and then HT-transformed coefficients are encoded by a SW-BPE encoder. The SW-BPE encoder includes the CCSDS BEP, performing bit plane encoding, and Slepian-Wolf encoder, encoding the coded bit plane. Slepian-Wolf encoder is implemented by QC-LDPC to generate the corresponding check bits, which are only transported into the compressed bit stream.

In decoding, the check bits combined with side information can be grouped together into new words to correct error. The side information can be obtained from BPE encoding HT-transformed coefficients of ${\hat{X}}_{i}^{\prime }$ which can be predicted from ${\hat{X}}_{i-1}$ reconstructed by spectral bans *X*_*i*−1_. The new code words after correcting the error and DC coefficients are combined into one to be decoded by using BPE to get the staging coefficients ${\hat{X}}_{i}^{s}$. The ${\hat{X}}_{i}^{s}$ and the HT-transformed coefficients of ${\hat{X}}_{i}^{\prime }$ carry out auxiliary reconstruction, inverse HT-transform, and inverse wavelet transform to the reconstructed ${\hat{X}}_{i}$. Since the bit streams after Slepian-Wolf encoding are only the check bits and not the bit plane bits, the high compression performance can be reached.

In the proposed algorithm, the side information is obtained by prediction from the previous band for the current band. This is the same as the traditional prediction-based approach and only prediction in decoder. In encoder, each band is independently encoded. This is the same as removing the traditional spectral prediction for multispectral image and can shift the complexity of encoder to the decoder. The strategy can reduce the complexity of encoder and is very suitable for the on-board application.

**Figure 4:**
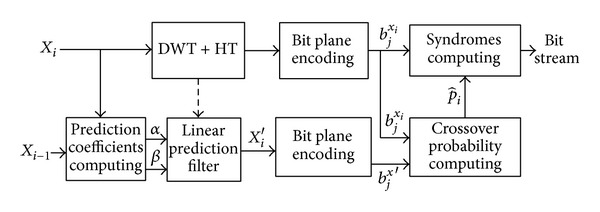
The encoding process of SW-BPE.

Figure 4 shows the encoding process of SW-BPE.

In Figure 4, *b* ^*w*^ denotes one bit plane of image *W*, *b*_*j*_^*w*^ denotes *j*th bit plane of image *W*, and *b*_*j*_^*w*^(*l*) denotes *l*th bit in the *j*th bit plane of image *W*. The encoding step is shown as in Algorithm 2.

**Figure 5:**
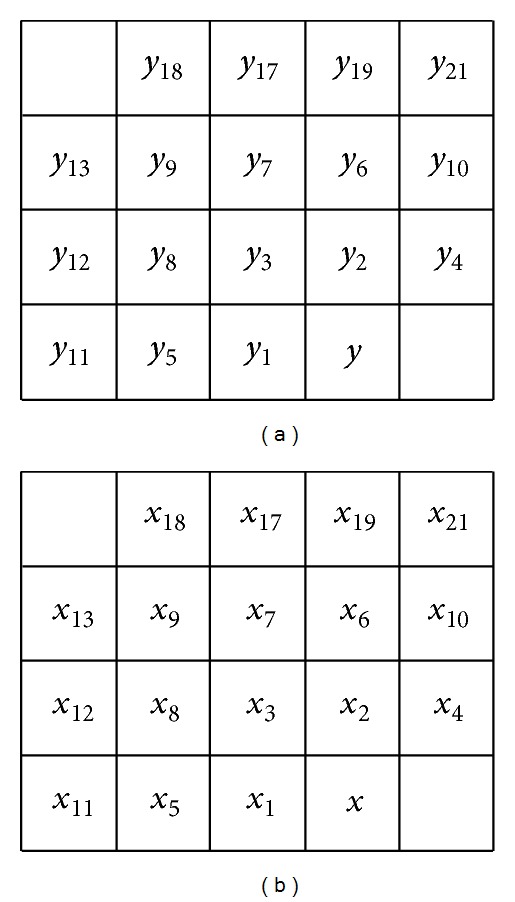
Two subimage windows, their pixels have the same spatial locations; (a) is a previous band and (b) is a current band.

In order to ensure the relationship of zero tree of bit plane, we use a first-order linear predictor to compute the coefficients as

(9) $$\begin{array}{c} x^{\prime }=\alpha x_{i-1}+\beta , \end{array}$$

where *x*_*i*−1_ denotes the pixels of previous band, which have the same spatial locations with the current pixels. In sensing of least minimum mean square error (LMMSE) for prediction error, the prediction parameters of the statistically optimal prediction can be expressed as

(10) $$\begin{array}{c} a=\frac{E\left\{x_{i}x_{i-1}\right\}}{\delta_{x_{i}}^{2}},\;\;b=m_{x_{i-1}}-am_{x_{i}}, \end{array}$$

where *m*_*x*_*i*−1__ = *E*{*x*_*i*−1_} is the expected values for the random variables *x*_*i*−1_ and *m*_*x*_*i*__ = *E*{*x*} is the expected values for the random variables *x*. In order to efficiently compute parameters *a* and *b* according to (10), we define two subimage windows, one is part of the current band, and another is part of the previous band. The pixels in the one window have the same spatial locations with another window, which is shown in Figure 5. The statistical parameter *a* can be approximated as

(11) $$\begin{array}{c} a=\frac{M\sum_{i=1}^{M}x_{i}y_{i}-\sum_{i=1}^{M}x_{i}\sum_{i=1}^{M}y_{i}}{M\sum_{i=1}^{M}x_{i}^{2}-{\left(\sum_{i=1}^{M}x_{i}\right)}^{2}}. \end{array}$$

Similarly, other parameter *b* can be likewise calculated.

In addition, we consider the QC-LDPC as the strategy of SW encoding because QC-LDPC is easy to hardware-implement. For fast and efficient coding, cyclic matrix *A* uses *b* × *b* permutation matrix as

(12) $$\begin{array}{c} P=\left[\begin{array}{ccccc} 0 & 1 & 0 & \cdots & 0\\ 0 & 0 & 1 & \cdots & 0\\ \vdots & \vdots & \vdots & \ddots & \vdots \\ 0 & 0 & 0 & \cdots & 1\\ 1 & 0 & 0 & \cdots & 0 \end{array}\right]. \end{array}$$

Consider *P* ^*j*^ is a permutation matrix, and it is obtained by unit matrix *P* ^*j*^ that shifts *i* times to the right. *i* is a positive integer. *P* ^*∞*^ is a zero matrix. The parity-check matrix can be constructed by *P* ^*j*^, which can be expressed as

(13) $$\begin{array}{c} P=\left[\begin{array}{ccccc} P^{a_{11}} & P^{a_{12}} & \cdots & P^{a_{1(k-1)}} & P^{a_{1k}}\\ P^{a_{21}} & P^{a_{22}} & \cdots & P^{a_{2(k-2)}} & P^{a_{2k}}\\ \cdots & \cdots & \cdots & \cdots & \cdots \\ P^{a_{j1}} & P^{a_{j2}} & \cdots & P^{a_{j(k-1)}} & P^{a_{jk}} \end{array}\right], \end{array}$$

where *a*_*il*_ ∈ {0,1,…, *q* − 1, *∞*}. The bit rate is *R* ≥ 1 − *j*/*k*. According to the bit rate, the constructed parity-check matrix can be expressed as

(14) $$\begin{array}{c} P=\left[\begin{array}{ccccccc} I & I & I & \cdots & I & \cdots & I\\ 0 & I & P & \cdots & P^{\left(j-2\right)} & \cdots & P^{\left(k-2\right)}\\ 0 & 0 & I & \cdots & P^{2(j-3)} & \cdots & P^{2(k-3)}\\ \cdots & \cdots & \cdots & \cdots & \cdots & \cdots & \cdots \\ 0 & 0 & \cdots & 0 & I & \cdots & P^{\left(j-1)(k-j\right)} \end{array}\right]. \end{array}$$

The bit rate can be determined by *j* and *k*. And the parity-check matrix can also be determined, and then coding is implemented. Since the elements of proposed parity-check matrix are 0 or 1 only and are computed by simple additions and shifts, QC-LDPC encoder is suitable for the hard implementation.

## 3. Experimental Results

In order to verify the feasibility and evaluate the proposed DSC combined with the CCSDS-IDC- (DSC-CCSDS-) based compression algorithm performance for multispectral images, we use several group multispectral remote sensing images. Each group of multispectral images is composed of three bands. In each group, the bit depth of each pixel is 8 bpp (bit per pixel). In our algorithm, each band performs 3-level two-dimensional Daubechies 9/7 DWT discrete wavelet transform and HT-transform, and each HT-transformed coefficient was quantized to 8 bits. The whole algorithm is implemented by MATLAB on a personal computer (PC). The working parameters of the PC are configured as: (1) two dual-core Intel CPU; (2) 3.6 GHz main frequency; (3) 4 GB of RAM.

**Figure 6:**
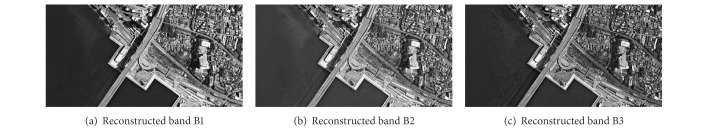
Encoding multiband images at 2.0 bpp.

Firstly, we verify the feasibility of our algorithm. A test multispectral image Akashi Kaikyō Bridge is used to test our approach. The multispectral test has three bands. The first band is denoted by B1, The second band by B2, and the third band by B3. Each band is 1325 × 678. The spectrum of B1 is 0.76–0.90 *μ*m, B2 is 0.63–0.69 *μ*m, and B3 is 0.52–0.60 *μ*m.  Figure 6 demonstrated the three reconstructed bands of the testing multispectral images. The B3 is considered as the key band. The compression encoding rate is set to 2.0 bpp. From the displayed images, the reconstructed images obtain better results because the proposed algorithm has a high signal-to-noise ratio.

Secondly, we evaluate the performance of our algorithm. To thoroughly evaluate our algorithm, a series of remote sensing multispectral images having different textures characteristics are used to perform compression testing. The testing experiment is performed at different compression code rates. The compression code rate is 0.25~4 bpp. In addition, we also use CCSDS-IDC algorithm to compress these multispectral images at different compression code rates. The tested compression results of the proposed CCSDS-DSC approach are compared with the CCSDS-IDC. We use the PSNR formula to evaluate the rate distortion performance of two compression approaches objectively. The PSNR can be expressed as

(15) $$\begin{array}{c} \text{PSNR}=10\text{lo}{\text{g}}_{10}\left(\frac{P}{\text{MSE}}\right)\;\left(\text{dB}\right),\\ \text{MSE}=\frac{1}{R\times C}\overset{R-1}{\underset{i=0}{\sum }}\;\overset{C-1}{\underset{j=0}{\sum }}{\left(x_{i,j}-x_{i,j}^{\prime }\right)}^{2}, \end{array}$$

where *P* is equal to 2^*B*^ − 1, *B* is the quantization bits of original images, *x*_*i*,*j*_ denotes the value of the pixel of original images at (*i*, *j*), *x*_*i*,*j*_′ denotes the value of the pixel of reconstructed images at (*i*, *j*), and *R* and *C* denote the number of row and column of multi-spectral images, respectively.

**Figure 7:**
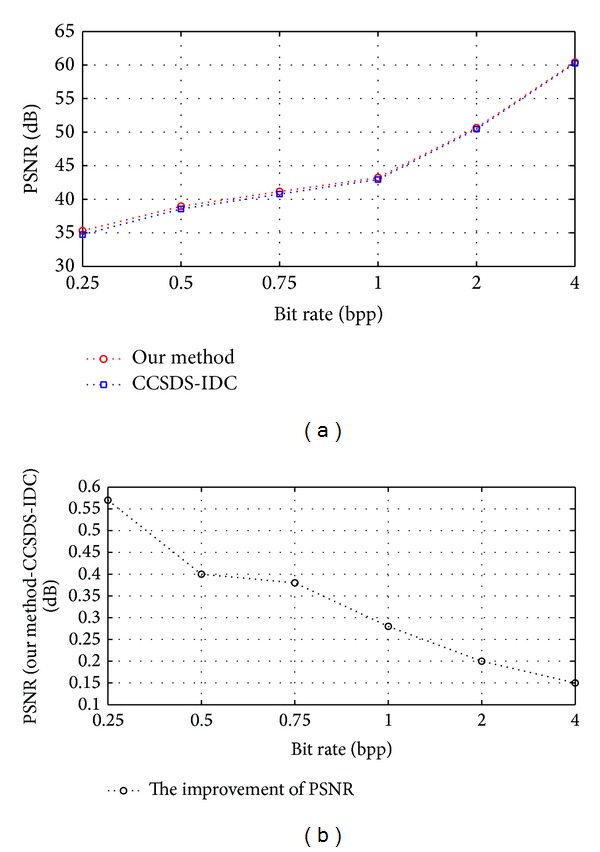
The test result of multiband images.

Figure 7 shows the comparison results of our algorithm and CCSDS-IDC approach. Because we apply the HT to DWT domain of each band and merge CCSDS-IDC to DSC by deep coupling way, the proposed algorithm has the better compression performance than CCSDS-IDC algorithm, and the PSNR improves 0.15~0.56 dB than the CCSDS-IDC compression codec in norm bit rate 2.0~0.25 bpp.

In [13], Zhang et al. proposed ViDis model remote sensing compression algorithm. In their testing results, the PSNR is 28.84 dB, 29.67 dB, 33.84 dB, and 38.83 dB at 0.4 bpp, 0.5 bpp, 1.0 bpp, and 2.0 bpp, respectively. They also compare their algorithm with JP2, DCQ, and GEO. Their algorithm improves 0.04 ~0.76 dB. In [14], Valsesia and Magli use CCSDS-123+rate Control B and CCSDS-122+POT+Reverse Warterfill compress SPOT5 multispectral images at 1.0 ~4 bpp. Their PSNR reaches 23.80~44.30 dB and 24.26~41.29 dB, respectively. In [15], adaptive transform + CSPIHT reaches 32.09~46.10 dB at 0.25~2.5 bpp. In [16], JPEG2000 MC, 3D SPECL, and LVQ-SPECK reach 22.44~34.54 dB, 24.41~41.53 dB, and 22.86~39.51 dB, respectively. Comparing the above compression algorithms with our approaches, our algorithm has the best compression performance.

The following compression times are only evaluations since our FPGA implementation of the CCSDS-DSC is not optimized. These evaluations are based on the lossy compression of an image of size 3072 × 128 at 1.0 bpp on a FPGA EVM board with system clock frequency at 88 MHz.  Table 1 demonstrates the comparison of complexity between the proposed multispectral compression algorithm and others.

From Table 1, the processing time of our algorithm reaches 0.013 *μ*s/sample; the data throughput is 76.9 MSPS, which is higher than JPEG2000 and CCSDS-IDC, so our approach has low complexity. In our project, the space CCD camera works at an orbits altitude of 500 km, the scroll angle of −40°~+40°, the latitudes of −70°~+70°, and the line working frequency is 7.2376~3.4378 kHz. In the line working frequency, capturing the image of 128 × 3072 requires 70.74 ms. Using our approach, the compressing the image of 128 × 3072 requires 5.12 ms. So, our approach can process the four bands images simultaneously. This meets the requirement of the project.

In addition, we use the XC2V6000-6FF1152 FPGA to implement the proposed algorithm. The design language is VerilogHDL, the development platform is ISE8.2, and synthesis tool is XST.  Table 2 demonstrates the occupancy of resources of our approach.

From Table 2, the LUT occupies 67%, Slices occupies 70%, and BRAM occupies 80%. Various indicators are lower than 95%, which meet the requirement of our project.

## 4. Conclusion

A DSC combined with the CCSDS-IDC compression algorithm is proposed for multispectral images in this paper. In our algorithm, first, each band is sparse represented by DWT to obtain wavelet coefficients. Then, the wavelet coefficients are encoded by BPE. Finally, the BEP is merged to the DSC strategy based on QC-LDPC by deep coupling way to remove the residual redundancy between the adjacent bands. A series of multispectral images is used to test our algorithm. Experimental results show that the proposed DSC combined with the CCSDS-IDC- (DSC-CCSDS-) based algorithm has better compression performance than traditional compression approaches in each band. To further reduce on-board encoding complexity, we will use a compressed sensing (CS) to take the place of CCSDS-IDC in our algorithm. We expect it will have a better compression performance for multispectral images.

## Figures and Tables

**Algorithm 1:**
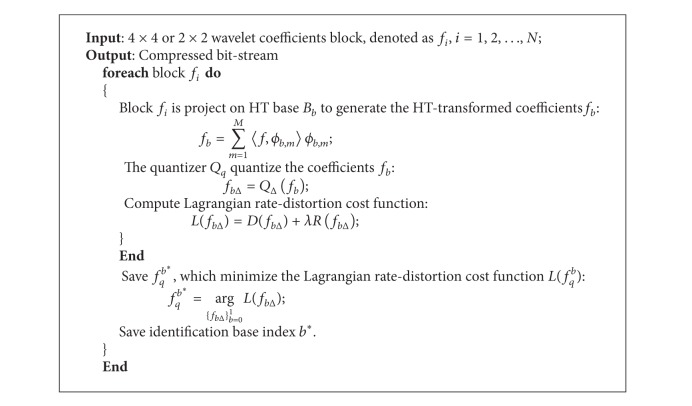
The HT-transform algorithm for one block.

**Algorithm 2:**
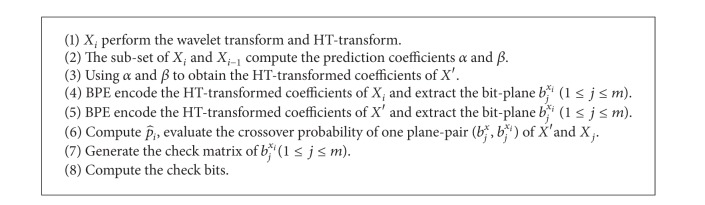
SW-BPE encoding process.

**Table 1:** The results of complexity comparison.

Methods	Times
Ours	0.013 *μ*s/sample
JPEG2000 [17]	0.11 *μ*s/sample
JPEG2000 [18]	0.04 *μ*s/sample
CCSDS-IDC [19]	0.08 *μ*s/sample
CCSDS-IDC [20]	0.05 *μ*s/sample
CCSDS-IDC [21]	0.025 *μ*s/sample

**Table 2:** The occupancy of resources.

Resources	Utilization rate
Slices	23655/33792 (70%)
4 Input LUTs	45281/67584 (67%)
BRAM	116/144 (80%)

## References

[1] Imani M, Ghassemian H (2014). Band clustering-based feature extraction for classification of hyperspectral images using limited training samples. *IEEE Geoscience and Remote Sensing Letters*.

[2] Pantazis M, van Gorp B, Robert O (2014). Portable remote imaging spectrometer coastal ocean sensor: design, characteristics, and first flight results. *Applied Optics*.

[3] Mukherjee K, Bhattacharya A, Ghosh JK, Aroraa MK (2014). Comparative performance of fractal based and conventional methods for dimensionality reduction of hyperspectral data. *Optics and Lasers in Engineering*.

[4] Ji R, Gao Y, Hong R (2014). Spectral-spatial constraint hyperspectral image classification. *IEEE Transactions on Geoscience and Remote Sensing*.

[5] Zhao H, Zhou P, Zhang Y (2013). Development of a dual-path system for band-to-band registration of an acousto-optic tunable filter-based imaging spectrometer. *Optics Letters*.

[6] Aggoun A (2011). Compression of 3D integral images using 3D wavelet transform. *IEEE/OSA Journal of Display Technology*.

[7] Nichols S, Kim H, Humos AA, Cho HJ A performance evaluation on DCT and wavelet-based compression methods for remote sensing images based on image content.

[8] Shapiro JM An embedded wavelet hierarchical image coder.

[9] Blanes I, Serra-Sagrista J (2010). Cost and scalability improvements to the Karhunen-Loêve transform for remote-sensing image coding. *IEEE Transactions on Geoscience and Remote Sensing*.

[10] Taubman D (2000). High performance scalable image compression with EBCOT. *IEEE Transactions on Image Processing*.

[11] Lu L, Zhang L, Zhang T (2013). JPEG2000-based optimization algorithm for effective compression display of remote sensing images. *Applied Mechanics and Materials*.

[12] Garcia-Vilchez F, Serra-Sagrista J (2009). Extending the CCSDS recommendation for image data compression for remote sensing scenarios. *IEEE Transactions on Geoscience and Remote Sensing*.

[13] Zhang Y, Cao H, Jiang H, Li B (2013). Visual distortion sensitivity modeling for spatially adaptive quantization in remote sensing image compression. *IEEE Geoscience and Remote Sensing Letters*.

[14] Valsesia D, Magli E (2014). A novel rate control algorithm for on board predictive coding of multispectral and hyperspectral images. *IEEE Transactions on Geoscience and Remote Sensing*.

[15] Bayazit U (2011). Adaptive spectral transform for wavelet-based color image compression. *IEEE Transactions on Circuits and Systems for Video Technology*.

[16] Dutra AJS, Pearlman WA, da Silva EAB (2011). Successive approximation wavelet coding of AVIRIS hyperspectral images. *IEEE Journal on Selected Topics in Signal Processing*.

[17] Leibo L, Chen N, Meng H, Zhang L (2004). A VLSI architecture of JPEG2000 encoder. *IEEE Journal of Solid-State Circuits*.

[18] Mathiang K, Chitsobhuk O Efficient pass-pipelined VLSI architecture for context modeling of JPEG2000.

[19] Huaichao W, Jun C, Xiaodong G High speed and bi-mode image compression core for onboard space application.

[20] Lin A, Chang CF, Lin CM High-performance computing in remote sensing image compression.

[21] Seo Y-H, Kim D-W (2007). VLSI architecture of line-based lifting wavelet transform for motion JPEG2000. *IEEE Journal of Solid-State Circuits*.

